# The Impact of the COVID-19 Pandemic on Orthopaedic Surgery Residency Applicants During the 2021 Residency Match Cycle in the United States

**DOI:** 10.5435/JAAOSGlobal-D-20-00103

**Published:** 2020-11-20

**Authors:** Nicholas C. Danford, Connor Crutchfield, Amiethab Aiyer, Charles M. Jobin, William N. Levine, T. Sean Lynch

**Affiliations:** From the Department of Orthopaedic Surgery, Columbia University Irving Medical Center, New York, NY (Dr. Danford, Mr. Crutchfield, Dr. Jobin, Dr. Levine, Dr. Lynch), and the Department of Orthopaedic Surgery, University of Miami/Miller School of Medicine, Miami, FL (Dr. Ayer).

## Abstract

**Introduction:**

The purpose of this study was to quantify the impact of the COVID-19 pandemic on rising fourth-year medical students' plans to apply to residency in orthopaedic surgery.

**Methods:**

We conducted a survey of rising fourth-year medical students. Primary outcome was the change in students' plans to apply to residency in orthopaedic surgery as measured by Likert scale response. Secondary outcomes were students' concerns about applying to residency during the pandemic.

**Results:**

A total of 462 students were planning to apply to residency in orthopaedic surgery. Women said that they were “less likely” to apply to orthopaedic surgery because of the pandemic (14.9% versus 5.5% of men, *P* < 0.001). Students identifying as Black/African American said that they were “less likely” to apply (16.9% compared with 8.8 of non-Hispanic White, *P* < 0.001). Students said that they had “somewhat fewer” or “many fewer” opportunities to get adequate exposure to orthopaedic surgery to make a specialty choice (88.9% of students).

**Discussion:**

We support the development of robust student advising and mentorship networks to address the uncertainty inherent in applying to residency during a global pandemic and curtail the racial and sex disparities discovered in this survey.

The outbreak of coronavirus disease (COVID-19) in late December 2019 in Wuhan, China, quickly developed into a worldwide pandemic that affected treatment of orthopaedic patients and education of medical trainees.^1,2,3,4^ As orthopaedic practices pivoted from a musculoskeletal focus to COVID-19 care to serve their communities, orthopaedic trainees were recruited or redeployed to staff overwrought emergency departments and intensive care units.^5,6,7^ The COVID-19 pandemic affected not only clinical care but also medical students applying to residency. Throughout the United States, cancellation of clinical rotations obviated opportunities for students to garner exposure to specialties of interest. The loss of this clinical exposure also has limited the capability for students to engage with faculty members, making it challenging to establish advising relationships that could potentially blossom into mentorships. In an effort to continue education efforts during the pandemic, medical schools have transitioned to online and distance learning.^8,9,10^

The in-person clinical rotation is a keystone of orthopaedic student training and is therefore difficult to replace with other forms of education.^11^ Orthopaedic residency applicants rely on these clinical rotations to demonstrate their interest in the field and to evaluate residency programs. The rotations offer attending and resident physicians the opportunity to kindle relationships with students in an attempt to identify future residents and potential mentees.^12,13^ Students who complete an away rotation at a given institution have an increased chance of matching with that institution compared with peers who do not complete the same away rotation.^12^ Thus, in the context of the COVID-19 pandemic, the inability for students to complete away rotations has changed the residency application processes.

We do not yet know how these changes will affect the application planning of the students applying to residency in orthopaedic surgery, inclusive of students of varying demographics. The purpose of this study was to quantify the impact of the COVID-19 pandemic on rising fourth-year medical students' plans to apply to residency in orthopaedic surgery. Our primary outcome was the change in students' plans to apply to residency in orthopaedic surgery as measured by Likert scale response. We also evaluated other effects of the pandemic, including the percentage of students with canceled or postponed clinical rotations and the percentage of students who felt a lack of exposure to the field and potential faculty/resident mentors.

Our hypothesis was that the COVID-19 pandemic would change the orthopaedic surgery residency application plans of rising fourth-year medical students, a finding that would be associated with sex, race, and geographic region in which a given student attends the medical school. We also hypothesized that in light of the pandemic, students would express concern about exposure to the field of orthopaedics and potential faculty/resident mentors within the field, which would cause them to consider taking an extra year before applying to residency.

## Methods

### Study Design and Participants

This is a prospective, cross-sectional survey study of medical students in the United States. Included participants were any person registered for a webinar that took place on May 20, 2020, who by survey identified as a rising fourth-year medical student with an expected graduation date of spring 2021. The purpose of the webinar was to provide guidance to potential applicants to orthopaedic surgery during the COVID-19 pandemic. Applicants applying to other fields were also invited to attend the webinar.

Exclusion criteria were any survey respondent who did not identify as a rising fourth-year medical student. We did not exclude survey respondents who declared that they were not applying to residency in orthopaedic surgery. We did our study according to the Strengthening the Reporting of Observational Studies in Epidemiology guidelines.^14^

### Bioethics

The study was conducted in strict accordance with bioethical principles. Institutional Review Board approval was obtained and documented.

### Data Collection

Demographic and outcome data were collected from survey responses through Qualtrics^XM^. The survey was distributed three times. First, on the morning of the webinar, an e-mail was sent to all registrants. Next, the survey was available through quick response code during the webinar. Finally, an e-mail was sent the next day to provide another opportunity for registrants to respond. Survey responses were anonymous.

### Survey Design

The survey was designed to respond to a novel world crisis, and thus, no validated survey was available. Survey design was based on consensus of an expert panel, a process that has precedent in the orthopaedic literature.^15^ Three attending physicians within the Department of Orthopaedic Surgery at one institution, all of whom have been educating and mentoring medical students from 6 to 23 years, comprised the expert panel. Participants could choose not to respond to any question.

### Variables

**Figure 1:**
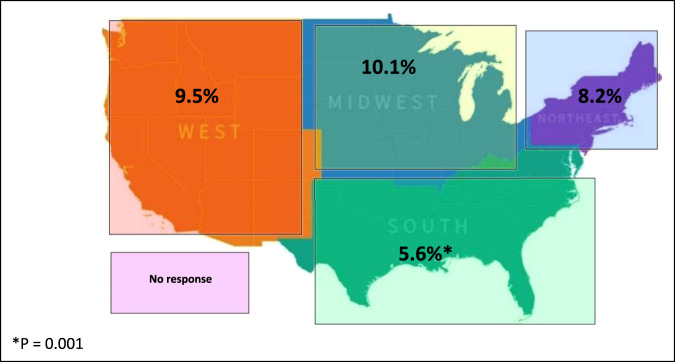
Figure demonstrating the percentage of students applying to orthopaedic surgery responding “less likely to apply” to the question “Has the COVID-19 pandemic impacted your decision to apply for a residency in orthopaedic surgery?”

The full survey featuring demographic and outcome variables is available in the supplementary materials section (see Supplemental Data File, http://links.lww.com/JG9/A103). Demographic variables were sex, race, region of medical school, (divided into West, Midwest, South, and Northeast) (Figure 1), and whether a participant was applying into orthopaedic surgery, dichotomized as yes or no. If a participant answered no, he or she selected from a drop-down menu another specialty. Outcome variables were responses measured using a Likert scale, dichotomous “yes” or “no” answers, or by free response where indicated to the remaining questions. The primary outcome variable was a Likert scale response to the question “Has the COVID-19 pandemic impacted your decision to apply for a residency in orthopaedic surgery?” with possible responses of “Much more likely to apply,” “More likely to apply,” “No change,” “Less likely to apply,” “Much less likely to apply,” and “I plan to apply but defer to a later cycle.”

### Statistical Analysis

Continuous variables were presented as the mean and the standard deviation, and categorical variables were presented as number and percentage. Likert scale responses were analyzed as continuous data.^16^ We used the chi-squared test, Student *t*-test, or analysis of variance test where appropriate to compare differences between students of different sex and race and from different regions of the country and students who were or were not applying to residency in orthopaedic surgery. Statistical analyses including geographical mapping were done using Qualtrics^XM^.

## Results

### Responses of All Students

Overall, 1,037 survey responses of 5,010 webinar registrants were noted, for a response rate of 20.7%. Of the respondents, 135 were excluded because they did not identify as rising fourth-year medical students with an expected graduation date of spring 2021. We therefore included 812 study participants. Of these, 462 (57.7%) responded that they were applying to residency in orthopaedic surgery. Three hundred eighty-nine (84.2%) of those who indicated they were applying to residency in orthopaedic surgery indicated that they were “definitely” applying. Compared with those not planning to apply to orthopaedic surgery, participants who said that they were planning to apply to residency in orthopaedic surgery were more likely to be men (71.2% versus 27.7%, *P* < 0.001). Race also varied significantly, with non-Hispanic White students, indicating that they were planning to apply to residency in orthopaedic surgery at a higher rate than those identifying with other races (*P* = 0.02). Geographic distributions were similar between students applying to residency in orthopaedics and students not applying in orthopaedics (*P* values for each region versus the rest of the country >0.05) (Table 1).

**Table 1:** Participant Demographics

Variable	Total	Applying to Residency in Orthopaedic Surgery^a^		
		Yes	No	*P* Value^b^
Sex	747	437	310	**<0.001**
Man	56.0%	71.2%	34.5%	
Woman	43.0%	27.7%	64.5%	
Race	748	437	311	**0.02**
Non-Hispanic White	57.2%	62.5%	49.8%	
Black/African American	6.7%	5.7%	8.0%	
Hispanic	7.9%	7.6%	8.4%	
Asian	20.9%	17.2%	26.0%	
Other	4.0%	3.9%	4.2%	
Prefer not to respond	3.3%	3.2%	3.5%	
Geographic location	762	446	316	
Northeast	28.1%	30.5%	24.7%	0.13
South	28.9%	28.9%	28.8%	0.64
Midwest	19.2%	19.1%	19.3%	0.95
West	10.8%	10.3%	11.4%	0.59
Outside the United States	0	0	0	

a Participants could skip a question at their discretion.

b *P* values <0.05 are considered significant and are presented in bold.

### Responses of Students Planning to Apply to Residency in Orthopaedic Surgery

The influence of the pandemic on an applicant's decision to plan to apply to residency in orthopaedic surgery was notably associated with sex, race, and geographic location in which a student attended medical school. Only 5.5% of men applying in orthopaedic surgery said that they were less likely to apply in orthopaedic surgery because of the pandemic, compared with 14.9% of women (*P* < 0.001) (Table 2). Regarding race, students planning to apply in orthopaedic surgery identifying as Black/African American said that they were “less likely” to apply to residency in orthopaedic surgery compared with students identifying as non-Hispanic White, Hispanic/Latino, Asian, or other (*P* < 0.001) (Table 3). Students identifying as Black/African American also said that they were “more likely” to apply to residency in orthopaedic surgery (8.0% Black/African American versus 1.5% of non-Hispanic White students, *P* < 0.001). Finally, students applying in orthopaedic surgery who attended medical school in the southern region of the United States were less likely to change their plans to “less likely to apply” to residency in orthopaedic surgery (South 5.6%, West 9.5%, Northeast 8.2%, and Midwest 10.1%, *P* = 0.001) (Figure 1).

**Table 2:** Response of Rising Fourth-Year Medical Students to the Question “Has the COVID-19 Pandemic Impacted Your Decision to Apply for a Residency in Orthopedic Surgery?” by Sex

Factor	Total	Planning to Apply to an Orthopaedic Surgery Residency			
		Yes		No	
		Man	Woman	Man	Woman
Total count	621	287	113	77	142
I am much more likely to apply to orthopaedic surgery	0.9%	1.9%	0.8%	0.0%	0.0%
I am more likely to apply to orthopaedic surgery	1.9%	1.9%	4.1%	0.9%	1.0%
No change	69.9%	81.4%^b^	66.1%	57.9%	62.0%
I am less likely to apply to orthopaedic surgery	6.2%	5.5%	14.9%^b^	3.7%	3.0%
I am much less likely to apply to orthopaedic surgery	3.6%	0.6%	3.3%	9.3%	5.0%
I am planning to apply to orthopaedic surgery but will defer to a later cycle	1.1%	1.0%	4.1%^b^	0.0%	0.0%

a Participants could skip a question at their discretion.

b *P* > 0.001.

**Table 3:** Response of Rising Fourth-Year Medical Students Applying to Orthopaedic Surgery to the Question “Has the COVID-19 Pandemic Impacted Your Decision to Apply for a Residency in Orthopedic Surgery?” by Race

Factor	Total^a^	Non-Hispanic white	Black/African American	Hispanic/Latino	Asian	Other
	397	262	25	29	68	13
I am much more likely to apply to orthopaedic surgery		0.8%	0.0%	0.0%	7.4%^b^	0.0%
I am more likely to apply to orthopaedic surgery		1.5%	8.0%^b^	3.4%	2.9%	0.0%
No change		86.3%^b^	76.0%	69.0%	80.9%	84.6%
I am less likely to apply to orthopaedic surgery		8.8%	16.0%^b^	13.8%^b^	4.4%	15.4%^b^
I am much less likely to apply to orthopaedic surgery		0.4%	0.0%	10.3%^b^	2.9%^b^	0.0%
I am planning to apply to orthopaedic surgery but will defer to a later cycle		2.3%	0.0%	3.4%^b^	1.5%	0.0%

a Participants could skip a question at their discretion.

b *P* > 0.001.

Regarding the question of taking additional time before residency, 137 students (33.3%) planning to apply to orthopaedic surgery said that they considered taking a year off, whereas 24 students (21.9%) not planning to apply to orthopaedic surgery said that they considered taking a year off (*P* = 0.002). Ninety students (91.0%) said that they would use this year for research.

Students voiced many concerns about the disruption to the application process. 88.9 percent of students planning to apply to orthopaedic surgery said that they had either “somewhat fewer” or “many fewer” opportunities to get adequate exposure to orthopaedic surgery to make a specialty choice (Table 4). 74.1 percent of students planning to apply to orthopaedic surgery said that they were either “very concerned” or “extremely concerned” about their ability to obtain meaningful letters of recommendation because of the pandemic. Away rotations were canceled for 82.8% of students, whereas 18.6% said that their home programs had not discussed logistical changes to away rotations, interviews, or the match caused by the COVID-19 pandemic.

**Table 4:** Applicants to Orthopaedic Surgery and Opportunities to Decide on Career Choice

How has the COVID-19 pandemic impacted your ability to get adequate exposure to orthopaedic surgery to make a specialty choice?	Total	411
	I have many fewer opportunities for exposure to orthopaedic surgery	50.9%
	I have somewhat fewer opportunities for exposure to orthopaedic surgery	38.0%
	No change	10.0%
	I have somewhat more opportunities for exposure to orthopaedic surgery	0.7%
	I have many more opportunities for exposure to orthopaedic surgery	0.5%

When asked about the relative importance of virtual opportunities in lieu of away rotations, students planning to apply to residency in orthopaedic surgery cited time spent in small groups or one on one with faculty and residents and learning about the hospital and facilities as either “very” or “extremely” important 90.9% of the time and 87.3% of the time, respectively.

## Discussion

Our study identifies important issues facing medical student orthopaedic education in the time of a worldwide crisis, including race and sex disparities and the need for focused advising, guidance, and mentorship for students. Our hypothesis that the COVID-19 pandemic would change fourth-year medical students' plans to apply to orthopaedic surgery, a finding that would be associated with sex, race, and geographic region in which a given student attends medical school was validated. Women were notably less likely to plan to apply to residency in orthopaedic surgery because of the pandemic, whereas Black/African American students were more likely to have a change in their application planning. Recent literature supports our data, suggesting that racial and sex disparities affect orthopaedic surgery training.^17,18,19^

Our hypothesis was also substantiated regarding student concerns about the pandemic and its effect on the residency application process. Students planning to apply to residency in orthopaedic surgery were concerned about many facets of the residency application process, such as opportunities to learn about other programs, ability to obtain meaningful letters of recommendation, and the choice of a year off before applying. Medical student advisors can use this information to guide discussion with potential applicants. Considering that 82.8% of students had away rotations canceled, virtual opportunities for students to learn about programs will be important. Our results suggest that students are willing to participate in on-line activities to garner more information about programs. Development of digitally based advising programs could further help students who do not have a home program or have had limited interaction with faculty at their institution. Such engagement could further amplify the strengths of a given program from a student perspective and could facilitate mentorship opportunities. We recognize that the mentorship is a longitudinal process, but endeavors of this type can certainly promote student advising at a minimum. Such opportunities could only help students, especially because 18.6% of students said that their home programs had not discussed logistical changes to the application process brought about by COVID-19.

We are only beginning to unravel the impact of the pandemic on medical education, with little available data amid scattered calls for change and adaptation.^10,20^ Moreover, other variables such as virtual as opposed to in-person interviewing have an unknown influence on the application and match process. To our knowledge, our study is the first that addresses and attempts to quantify this impact. The orthopaedic residency application progress is competitive.^21^ Students seek strong mentorship, especially in times of uncertainty. Based on our data, we support further efforts to strengthen education and mentorship efforts for students of different sexes and/or of differing racial backgrounds.

Our study has numerous strengths. We have a large sample size of students well-distributed geographically. The average number of applicants to orthopaedic surgery per year for the past five years was 1,262, indicating that with 462 participants identifying as fourth-year medical students planning to apply to residency in orthopaedic surgery, we captured approximately 36.6% of the applicant pool.^22^

Our study has certain weaknesses. Our data capture student planning for the application process and therefore do not capture whether a given student pursued a given action (for example, whether a student applied to residency in orthopaedic surgery after responding to the survey that he or she would apply to residency in orthopaedic surgery). Despite this limitation, we find our data valuable because it exposed student planning at an important point of the application cycle, which can guide mentorship. In addition, previous data show that orthopaedic applicant response to surveys reflects data accrued by the National Residency Matching Program.^21^

Another weakness is our lack of a control group. Instead, our comparison groups were among differing subsets of the medical student population and among those who identified as either planning to apply or not apply to orthopaedic surgery. A historical control group specifically would aid in analysis because it would show whether previous trends regarding applicant demographics are similar or different from those revealed by our survey. Our study also has a potential selection bias because students who were more likely to be concerned about the effect of the pandemic on their application may be more likely to attend the webinar. A better study population would include every rising fourth-year medical student who before the pandemic had declared that he or she would apply in orthopaedic surgery. However, because of the large sample size of 462 of an anticipated 750 applicants per program during a typical application cycle, we do not consider this detrimental to interpreting our results. Another weakness is that we did not collect other demographic data, such as socioeconomic status, that also can influence residency decision-making.

In conclusion, based on our data, we support the addition of robust mentorship networks to address the uncertainty inherent in applying to orthopaedic residency during a global pandemic and to curtail the racial and sex disparities uncovered by this survey.
